# Immune responses to *Neisseria gonorrhoeae* and implications for vaccine development

**DOI:** 10.3389/fimmu.2023.1248613

**Published:** 2023-08-17

**Authors:** Thomas Belcher, Christine S. Rollier, Christina Dold, Jonathan D. C. Ross, Calman A. MacLennan

**Affiliations:** ^1^ Jenner Institute, Nuffield Department of Medicine, University of Oxford, Oxford, United Kingdom; ^2^ School of Biosciences, University of Surrey, Guilford, United Kingdom; ^3^ The Oxford Vaccine Group, Department of Paediatrics, University of Oxford, Oxford, United Kingdom; ^4^ Sexual Health and HIV, University Hospitals Birmingham NHS Trust, Birmingham, United Kingdom

**Keywords:** immunology, gonococcus, Neisseria, STI, vaccines, gonorrhea

## Abstract

*Neisseria gonorrheoae* is the causative agent of gonorrhea, a sexually transmitted infection responsible for a major burden of disease with a high global prevalence. Protective immunity to infection is often not observed in humans, possible due to high variability of key antigens, induction of blocking antibodies, or a large number of infections being relatively superficial and not inducing a strong immune response. *N. gonorrhoeae* is a strictly human pathogen, however, studies using mouse models provide useful insights into the immune response to gonorrhea. In mice, *N. gonorrhoea* appears to avoid a protective Th1 response by inducing a less protective Th17 response. In mouse models, candidate vaccines which provoke a Th1 response can accelerate the clearance of gonococcus from the mouse female genital tract. Human studies indicate that natural infection often induces a limited immune response, with modest antibody responses, which may correlate with the clinical severity of gonococcal disease. Studies of cytokine responses to gonococcal infection in humans provide conflicting evidence as to whether infection induces an IL-17 response. However, there is evidence for limited induction of protective immunity from a study of female sex workers in Kenya. A controlled human infection model (CHIM) has been used to examine the immune response to gonococcal infection in male volunteers, but has not to date demonstrated protection against re-infection. Correlates of protection for gonorrhea are lacking, which has hampered the progress towards developing a successful vaccine. However, the finding that the *Neisseria meningitidis* serogroup B vaccines, elicit cross-protection against gonorrhea has invigorated the gonococcal vaccine field. More studies of infection in humans, either natural infection or CHIM studies, are needed to understand better gonococcal protective immunity.

## 1 Introduction

*Neisseria gonorrhoeae* is a human-restricted Gram-negative bacterium responsible for infections of the genital tract as well as the rectum, the pharynx, and the eyes. Collectively these infections are known as gonorrhea and are primarily spread through sexual contact. Infection of the lower genital tract can range from asymptomatic to more invasive ascending infection. In women, this can be particularly severe and can result in pelvic inflammatory disease (PID), ectopic pregnancy and infertility (1). Severe outcomes of gonorrhea are particularly problematic in low- and middle-income countries (LMICs), where *N. gonorrhoeae* is a major cause of infertility (2). In addition, co-infections with human immunodeficiency virus (HIV) are common, and infection with *N. gonorrhoeae* increases the risk of transmission and acquisition of HIV infection (3).

There is an increasing prevalence of antimicrobial resistant (AMR) among *N. gonorrhoeae*. Strains that are resistant to the current treatment options have been characterized, including strains resistant to both ceftriaxone and azithromycin (4, 5), making research into alternate therapies as well as vaccines a priority. Moreover, reinfection following natural infection is common in humans, and broad protective immunity against multiple strains of gonococcus is not induced (6–8). A study from the UK of patients with gonorrhea showed that prior infection with *N. gonorrhoeae* was the strongest predictor of current infection (9). The association between previous infection and current infection was only true for prior gonococcal infection and not other STIs, suggesting that this is not just a consequence of high risk behavior. Furthermore, up to one third of gonorrhea patients in England have had some prior gonococcal infection (9). Since repeat infections are common and cannot completely be accounted for by high risk behavior, it appears that infection does not often lead to protective immunity.

What is not clear is whether the lack of protective immunity is due to immune evasion by the bacteria, the failure to mount the correct type of immune response that results in protection, the antigenic variation displayed by gonococcus, or a combination of factors. Recently, outer membrane vesicle (OMV) vaccines against capsular group B *Neisseria meningitidis* (MenB) was shown to cross-protect against *N. gonorrhoeae*. This finding suggests that it is possible to induce protection against gonococcus, reigniting efforts to develop a specific vaccine for gonorrhea (10–12). Further investigation is needed as both the antigens involved and the immune mechanisms of protection are unclear. There is a need to understand more about the natural immune response to gonococcus, why protective immunity is often not induced and why previous vaccine candidates have failed. Further knowledge is also required about the natural human immune response to *N. gonorrhoeae* infection, including whether there are individuals who do mount a protective immune response, either to the infecting strain or to heterologous strains. Identifying correlates of protection would help to clarify what immune response should be elicited by a candidate vaccine.

**Figure 1:**
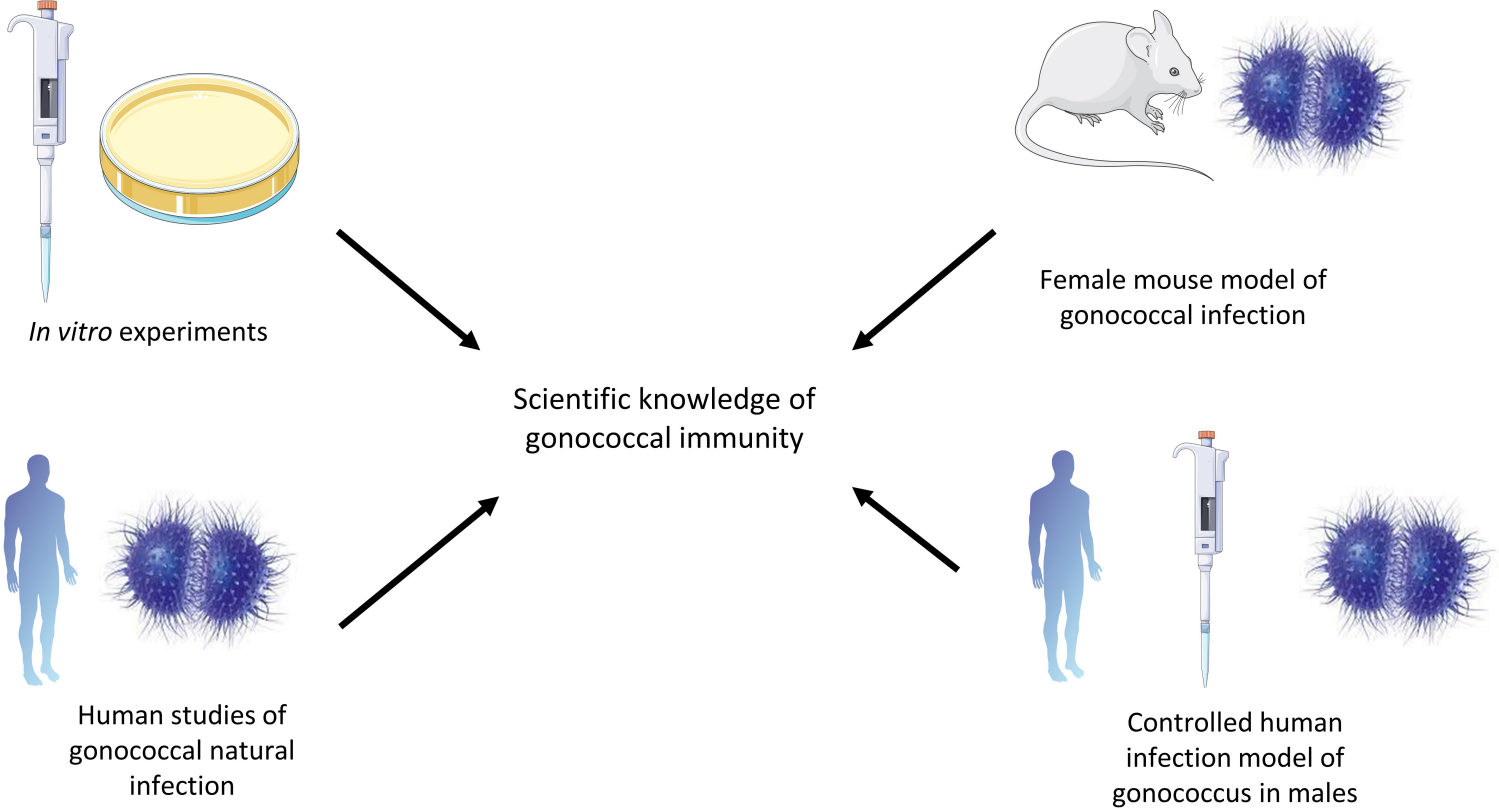
Different methods that have contributed to the field of gonococcal immunity. *In vitro* studies have been valuable in describing the interactions between gonococci and complement, for example. The murine female genital tract model is a transient infection model but is useful in describing the immune responses to gonococci and evasion mechanisms. Human studies of natural infection are valuable in studying gonococci in their natural host, while the controlled human infection model of gonococci can be used to describe the immune response to gonococci in the natural host in a controlled way, but only in males in a transient way.

This article summarizes current knowledge regarding immunity to *N. gonorrhoeae*, from *in vitro* studies, the murine female genital tract model of gonococcal infection, human studies of natural infection and the controlled human infection model (**Figure 1**). Furthermore, we discuss current knowledge gaps that need to be addressed to facilitate vaccine design.

## 2 Gonococcal biology and immunity

### 2.1 Variability of key antigens

*N. gonorrhoeae* has evolved many different routes to evade the human immune response. *N. gonorrhoeae* is a highly antigenically variable pathogen. It is naturally competent at taking up DNA from the environment, which may then be integrated into the genome by homologous recombination (13). This means variation between strains is a relatively common occurrence, and gives rise to populations which are not clonal (13–15). Gonococci are also subject to phase variation, and expression of specific genes is reversibly switched on or off leading to an additional source of variation between and within strains. Phase variation regulates the expression of *opa* genes, for example, which code for outer membrane proteins in *N. gonorrhoeae*. A single gonococcus can possess up to 11 different *opa* genes, all under phase variation due to differences in the number of CTCTT repeats in each gene caused by slipped-strand mispairing (13, 16, 17). In addition, a number of genes which encode glycosyltransferases (*lgt*) are subject to phase variation (18, 19). Expression of these genes results in differentially glycosylated lipooligosaccharide (LOS) on the surface of the bacteria. Gonococcal LOS has been shown to be immunogenic, and is the basis for a peptide mimetope candidate vaccine modelled on the 2C7 LOS epitope (20). It is possible that the expression of different glycosylation patterns of LOS are an immune evasion strategy, since mutant strains in which the expression of *lgt* genes is fixed are differentially bound and killed by a monoclonal antibody targeting 2C7 (21).

The genome of *N. gonorrhoeae* also contains several genes encoding pili, an adhesin mostly made up of PilE. Other copies of these genes, called *pilS*, are transcriptionally silent since they have no promoter (22). Each of these genes is variable in sequence, which results in different amino acid sequences in surface-exposed regions of the protein. Variation in *pilE* takes place by gene conversion (23, 24), which allows for the transfer of the DNA sequence from one donor *pilS* to target *pilE*, changing the sequence of the latter without changing the former. An efficacy study of a candidate vaccine based on purified pili failed possibly due to the variable nature of gonococcal pili (25).

PorB is an outer-membrane porin which can be used as a basis for gonococcal typing with one strain expressing one *porB* type. However, there is extensive variation in *porB* sequences between strains (26, 27). As gonococcus is a highly variable pathogen, especially within immunogenic antigens, a successful vaccine needs to consider worldwide strain coverage as well as account for continued evolution over time. The search for protective antigens that provide universal coverage in the face of a highly variable pathogen continues. It may be that there is a need for a reverse vaccinology approach to developing a successful gonococcal vaccine, as was the case for group B meningococcus (28). The specific antigens responsible for the cross-protective effect mediated by the Men B vaccine formulations are still to be identified (10, 11).

### 2.2 Evasion of complement

*N. gonorrhoeae* has developed many mechanisms to resist complement, suggesting a strong evolutionary pressure to do so. Firstly, gonococcus can recruit complement inhibitors. Gonococcal PorB, particularly the PorB1a allele, can bind the complement inhibitor C4BP, which correlates with the ability to evade complement-dependent killing (29, 30). Another complement inhibitor, factor H, can also be bound by PorB (31, 32). C4BP- and factor H-binding by gonococcus are human-specific. The gonococcal adhesin OpaA can bind the complement inhibitor vitronectin, which has been proposed to increase resistance to complement (33), and Opa proteins have been linked to serum resistance (34). In addition, *N. gonorrhoeae* has the ability to resist killing by normal human serum by scavenging CMP-Neu5Ac in order to sialylate lipo-oligosaccharide (LOS) (35, 36), which leads to a reduction in binding of anti-gonococcal antibodies and deposition of C4b (37, 38).

The importance of complement in immunity to *N. gonorrhoeae* can be inferred from studies indicating that individuals with primary immunodeficiencies of terminal complement components, who are unable to form the C5b-9 membrane attack complex, are at increased risk of disseminated gonococcal infection (39, 40). Though correlates of protection against gonorrhea are not known, this finding implies that antibody-dependent complement-mediated killing is an important mechanism of protection, at least in preventing invasive disease. Whether complement is involved in preventing more localized infection is not known. The finding that protection against meningococcal disease induced by meningococcal vaccines correlates with bactericidal antibody levels also supports the concept that complement is generally an important component of the immune response to genus *Neisseria* (41).

*N. gonorrhoeae* is able to evade immunity through the production of antibodies which block the action of protective antibodies targeting other antigens. An IgG antibody present in convalescent serum following gonococcal infection was found to block serum bactericidal activity against *N. gonorrhoeae* (42). This inhibitory activity is not the result of preventing the binding of complement since adding blocking antibody increases deposition of C3 and C5b-9, but due to deposition of complement at sites that does not lead to killing (42). These blocking antibodies were shown to bind bacterial target protein III (PIII), now called reduction modifiable protein (Rmp), which is present in all gonococcal strains and is highly conserved (42, 43). Antibodies to purified Rmp block killing in bactericidal assays, while depleting serum of anti-Rmp antibodies increases killing of *N. gonorrhoeae* by immune serum (43).

This finding was used to explain why bactericidal activity in convalescent serum from patients following disseminated gonococcal infection is usually very low despite high antibody levels (43). In a longitudinal study of 243 female sex workers in Nairobi, Kenya, those with anti-Rmp antibody were at increased risk of infection compared with those who lacked anti-Rmp antibody (44). Whether there is a need to remove Rmp from candidate whole cell *N. gonorrhoeae* vaccine strains is not known, but it is clear that induction of anti-Rmp antibodies can block the function of antibodies to other antigens. Serum from a mouse immunized with a mutant strain of *N. gonorrhoeae* lacking Rmp was more bactericidal than serum taken from a mouse immunized with the corresponding wild-type strain (45). Mice given anti-Rmp antibody by passive transfer or immunized intraperitoneally with recombinant Rmp, show delayed clearance of gonococcus following administration of anti-LOS bactericidal monoclonal antibody 2C7, as well as increased bacterial loads (46). In this study, anti-Rmp antibodies led to a reduction in the number of C3 molecules deposited on gonococci, in contrast to a previous study, which showed that anti-Rmp antibody increased deposition of C3 on cells but reduced killing (42). Together, these findings suggest that anti-Rmp antibodies can block killing of gonococci.

### 2.3 Evasion of bacterial killing by phagocytosis

*N. gonorrhoeae* infection induces a local immune response characterized by the infiltration of neutrophils to the site of infection, which is insufficient for clearance due to the ability of gonococci to evade phagocytosis by neutrophils and neutrophil extracellular traps (NETs) (47, 48). Mechanisms for this include failure to be taken up by neutrophils due to expression of pili (49) and resistance to intracellular killing (50, 51) which can be overcome by opsonophagocytosis following the addition of complement and serum (52). Gonococcus entering neutrophils via Opa binding to CEACAM1 or CEACAM6 causes inhibition of a pro-inflammatory response and neutrophil effectors (53, 54). Alternatively, the bacteria can adhere to the uropod of neutrophils to hide from their phagocytic activity (55).

Gonococcus can evade killing by neutrophils through modifying its peptidoglycan or LOS (56, 57), as well as resist killing by antimicrobial peptides through expression of the MtrCDE efflux pump (58, 59) and inhibit lysozyme killing (60). Gonococcus is also is able to degrade NETs through secretion of a nuclease (61) as well as inhibiting the maturation of the neutrophil phagosome, involving the pilus and PorB (48, 62). Further evasion of phagocytic killing by neutrophils takes place via binding to C4BP or by entering the neutrophil through CR3 inducing so-called ‘silent phagocytosis’ and reduced killing (63, 64).

*N. gonorrhoeae* can also evade killing by macrophages. Gonococci are phagocytosed by macrophages and while many of the bacteria are killed, some are able to survive and even replicate inside macrophages (65). It has been suggested that this is because *N. gonorrhoeae* is able to prevent the maturation of the phagosome, possibly mediated by PorB (65, 66). Furthermore, gonococci can prevent apoptosis in macrophages, suggesting preservation of a reservoir of bacteria for future replication (65). Additionally, *N. gonorrhoeae* can stimulate macrophages to take on an M2 phenotype associated with immunosuppressive functions (67). Gonococcal infection of M0 macrophages *in vitro* induces expression of M2 markers CD163 and CD206, but is not associated with the M1 markers CD86, MHCII, TLR-4 or CD64. IL-10 secretion occurs from macrophages stimulated with gonococci (67). Taken together, these findings suggest that gonococci induce a suppressive phenotype in macrophages, that increases the capacity for *N. gonorrhoeae* to survive after phagocytosis, but may also contribute to wider immune suppression through reduced capacity for antigen presentation and secretion of IL-10. For a detailed review of the interactions between *N. gonorrhoeae* and macrophages see Escobar et al. (68).

## 3 Mouse models

Despite the human restriction of *N. gonorrhoeae*, female BALB/c mice can be infected vaginally with gonococcus after pretreatment with 17-β-estradiol and the use of antibiotics to prevent the overgrowth of natural flora. Inoculation with 10^6^ colony forming units (CFU) has resulted in a mean duration of infection of 12.8 days (69). The mouse model of gonococcal infection is therefore imperfect, since duration of infection is limited. The use of hormones and antibiotics to make the mouse permissible to gonococcal infection also sets this model apart from human infection. Nevertheless, this approach allows colonization and bacterial replication to take place since higher numbers of gonococci are recovered from mice than are administered (69). Following infection of mice in the 17-β-estradiol model, gonococci localize in the vaginal and cervical tissue where a local inflammatory response is induced, characterized by infiltration of macrophages and neutrophils (70), similar to human infection (48). Murine gonococcal infection induces a specific antibody response both in the genital mucosa and plasma, but this is transient and not sufficient to prevent reinfection with the same strain (70). The mouse model is of value for studying the immune response to *N. gonorrhoeae* with the caveats that gonococcus does not cause ascending or disseminated infection in mice, infection is quite transient, and human-specific immune evasion mechanisms exist for *N. gonorrhoeae* which cannot be studied in wild-type mice.

The 17-β-estradiol mouse model has permitted the discovery of mechanisms by which *N. gonorrhoeae* directly evades induction of a protective immune response. The immune response to *N. gonorrhoeae* in the model is Th17 polarized, driven by the induction of IL-17 and characterized by an influx of neutrophils into the genital tract (71). In early studies, blocking IL-17 led to prolonged infection, suggesting that IL-17 and the Th17 response play a role in the clearance of gonococcus. Subsequently, the Th17 response was found to be partially dependent on expression of TGF-β, which in turn suppressed Th1 and Th2 responses (72). Blocking TGF-β accelerated the clearance of *N. gonorrhoeae*, allowing for the induction of Th1 and Th2 responses, leading to the development of anti-gonococcal antibodies and enhanced resistance to reinfection. The use of mice with targeted immune gene deletions of IL-12 (Th1-deficient) or IL-4 (Th2-deficient) showed that accelerated clearance of infection was Th1-dependent while resistance to reinfection required both Th1 and Th2 responses. Therefore, the current understanding is that *N gonorrhoeae* actively promotes a Th17 response which suppresses Th1 and Th2 responses needed for protective immunity in mice. The Th17 response to *N. gonorrhoeae* was shown to be induced by gonococcal LOS signaling through TLR4, while induction of TGF-β at least partially depended on expression of gonococcal Opa, since incubation of spleen cells with an Opa-deficient mutant resulted in a lower production of TGF-β (73). In summary, *N. gonorrhoeae* induces a Th17 response in mice, which can result in bacterial clearance, but which is suboptimal for protection compared to Th1 responses and does not result in protective immunity. Through the induction of TGF-β, the Th17 response suppresses the Th1 and Th2 responses needed for accelerated clearance and protection from reinfection.

**Figure 2:**
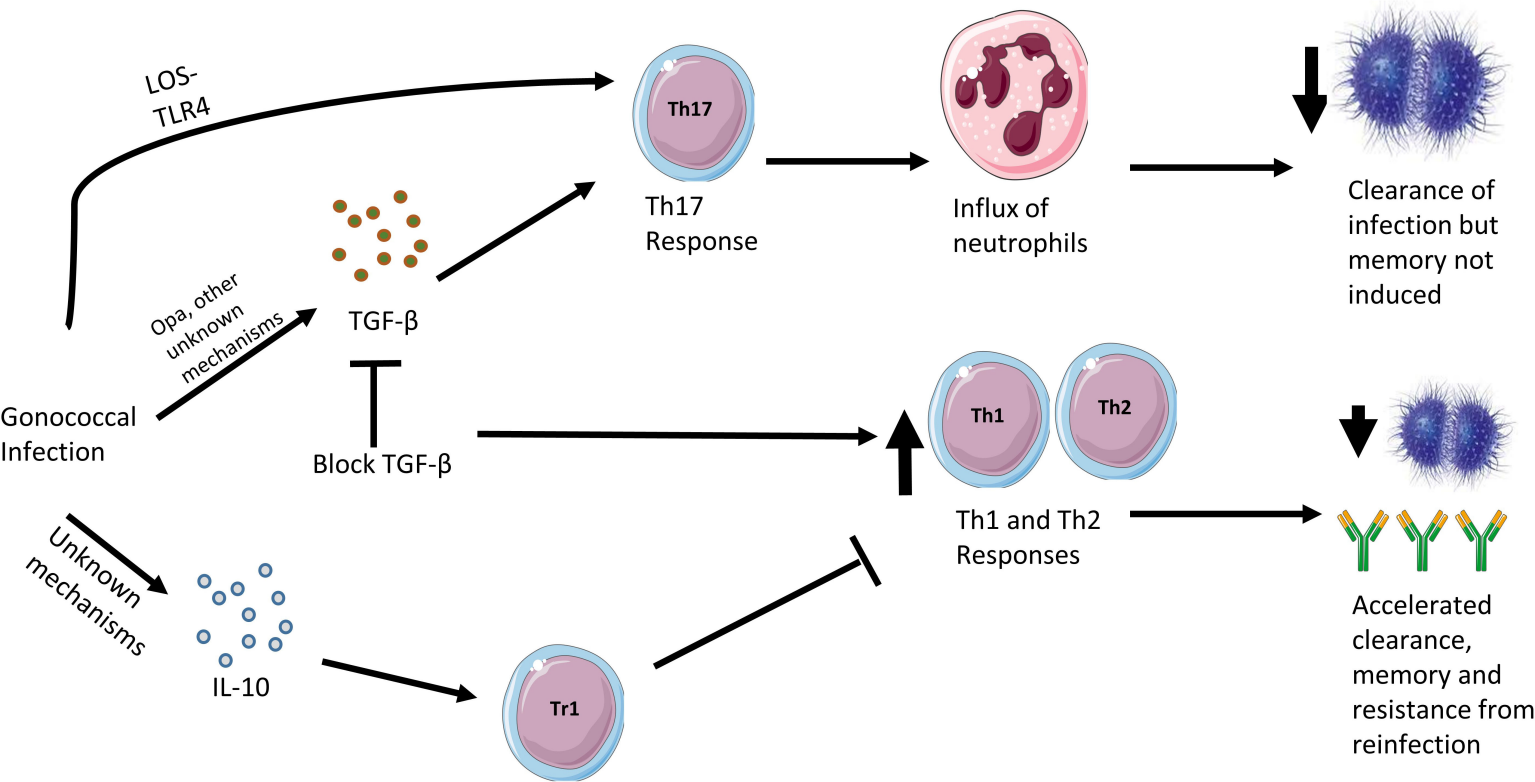
Cytokine responses in the murine female genital tract model of gonococcal infection. Gonococcal infection in the female mouse leads to a mostly Th17 response, driven by interactions between gonococcal LOS and murine TLR4 as well as stimulation of TGF-β, which is partly driven by gonococcal Opa surface proteins. The consequences of a Th17 response is an influx of neutrophils in the murine genital tract and clearance of the gonococci. However, if TGF-β action is blocked, Th1 and Th2 responses are observed, which leads to accelerated clearance, memory and resistance to reinfection. On infection with gonococci, the female mouse also responds by secreting IL-10 through an as yet undiscovered mechanism. This stimulates the differentiation of Tr1 cells and the inhibition of protective Th1 and Th2 responses.

Gonococcal infection in the 17-β-estradiol mouse model also induces expression of IL-10 leading to the expansion of type 1 regulatory T cells (Tr1 cells) (74). Blocking IL-10 and Tr1 cells leads to an increased Th1 and Th2 response, accelerated clearance and protection from reinfection, with increased levels of anti-gonococcal antibodies. Therefore, it appears that *N. gonorrhoeae* has evolved two distinct mechanisms: induction of IL-10 and TGF-β, in order to suppress a protective Th1 and Th2 immune response. This may explain why the immune response to natural infection does not result in protection to reinfection, although it is unclear whether these findings apply to human infection. A summary of the cytokine responses to gonococcal infection in the female mouse model is shown in **Figure 2**.

Co-administration of *N. gonorrhoeae* and encapsulated IL-12 directly in the vagina (simulating a Th1 response) leads to accelerated clearance and protection from reinfection with increased anti-gonococcal antibodies (75). Protection against secondary reinfection extends to heterologous strains and persists for at least 6 months (76). The protective immunity induced by IL-12 co-administration is dependent on IFN-γ and B cells, suggesting that these are important for protection against *N. gonorrhoeae*. A further study found that co-administration of microencapsulated IL-12 and outer membrane vesicles from *N. gonorrhoeae *via the intranasal route accelerates clearance in a murine gonococcal challenge model (77). This co-administration leads to an increase in levels of serum IgG, salivary IgA and vaginal IgG and IgA anti-gonococcal antibodies as well as IFN-γ production from CD4^+^ T cells isolated from the iliac lymph nodes. Importantly, protection against heterologous strains of gonococcus was observed. MAP1, a vaccine candidate based on a peptide mimic of a conserved gonococcal LOS epitope (2C7), induces a Th1 polarized immune response in BALB/c mice and bactericidal anti-LOS antibodies (78). Immunization with MAP1 shortens carriage of *N. gonorrhoeae* and lowers bacterial burden, effects that can be achieved with passive administration of a monoclonal antibody to 2C7 (mAb2C7) (78). The protective mechanisms which reduce pathogen burden and length of infection in response to mAb2C7 administration are dependent on complement (79). While mAb2C7 is functional in neutrophil-depleted mice and mice with blocked C5a receptors, the protective effects are lost in C1q null mice, C9 null mice or in mice in which C5 function has been blocked. Furthermore, a version of mAb2C7 with amino acid substitutions resulting in reduced binding to C1q was ineffective at killing gonococci *in vitro*. Therefore, complement appears to be essential for protective immunity to gonococcus in mice, through antibody-directed complement-mediated killing. A vaccine candidate based on a tetrapeptide mimic of the 2C7 epitope was effective in accelerating clearance and reducing colonization in BALB/c mice, and stimulated production of bactericidal specific IgG (20).

## 4 Human immunology

### 4.1 Natural infection

In contrast to immunological studies in mice, which can be performed in a highly controlled way, human studies have largely been restricted to measuring the immune response to natural infection. Different studies on the human immune response often include cohorts of varying sizes and backgrounds, while measuring different types of samples often taken at different time-points post-infection. This makes comparisons of individual studies difficult. While there is much still to be understood about the immune response to natural gonococcal infection, a lot of valuable insight has already been gained by such studies. A summary of the studies of natural infection of gonococcus in humans is shown in **Table 1**.

**Table 1:** Studies of natural infection of humans with *Neisseria gonorrheoae* detailing subjects, methods and key findings.

Reference	Authors	Year	Population/disease state	Methods	Findings
(80)	Kasper et al.	1977	5 women and 1 man with no history of gonococcal infection; 13 men with uncomplicated infection; 22 women with uncomplicated infection; 19 women with PID; 1 man with gonococcal epididymitis	Bactericidal antibody titre	Prolonged carriage of gonococcus and severity of disease correlates with development of bactericidal activity
(81)	Hook et al.	1984	8 patients with disseminated gonococcal infection; 1 patient with gonococcal epididymitis; 4 patients with gonococcal salpingitis; 2 volunteers with no history of gonococcal infection; 1 patient with acute meningococcemia. All were of unspecified gender	Western blot of sera to gonococcal antigens; bactericidal activity	Serum from nearly all gonococcal patients reacted with Por, most reacted with LPS by Western blot; bactericidal activity was partially, but no wholly due to antibodies targeting LPS
(82)	Buchanan et al.	1980	15 women with gonococcal PID	Serotyping for principal Por-type	Gonococcal PID induces some immunity to repeated episodes of PID of the same Por-type
(83)	Plummer et al.	1989	227 female commercial sex workers	Serotyping for principal Por-type	Por-types infecting the population changed over time; subjects who were HIV-uninfectedwere less likely to have gonococcal infections; frequency of gonococcal infection was inversely related to duration of prostitution; subjects were less likely to be re-infected with the same por-type
(84)	Hedges et al.	1999	120 male and female attendees at an STI clinic, infected with gonococcus and not	Antibody ELISA to formaldehyde-fixed gonococci on serum and genital secretions (IgG, IgA and IgM)	Both serum and mucosal anti-gonococcal antibodies were only modestly higher in subjects infected with gonococcus than in control subjects; previous history of gonococcal infection did not affect levels of gonococcal-specific antibodies
(85)	Hedges et al.	1998	66 female attendees at an STI clinic, infected with gonococcus and uninfected	Levels of IL-1, IL-6 and IL-8 in cervical mucus and serum by ELISA; Levels of IL-10 and TGF-β in cervical mucus and vaginal washes	Mucosal or serum cytokine levels were not different in gonococcus-infected subjects compared to uninfected controls, except for serum IL-6, which was elevated in gonococcus-infected subjects.
(86)	Gagliardi et al.	2011	27 gonococcus-infected patients (26 male, 1 female) varying levels of gonococcal disease; 17 male healthy controls	Serum concentrations of IL-17, IL-23 and IFN-γ	Serum concentrations of cytokines tested were significantly higher among gonococcus-infected subjects compared to controls; among those gonococcus-infected subjects there was an inverse correlation between levels of IL-17 and IFN-γ
(87)	Masson et al.	2015	317 women, gonococcus-infected and uninfected	Concentrations of up to 42 cytokines in mucosal samples or plasma by multiplex; Th17 response by intracellular cytokine staining of IL-17 in PBMCs	IL-17 levels were higher in women who had gonococcal infections, but this association was not significant after accounting for co-infections
(88)	Mensforth et al.	2020	405 gonococcal patients at an STI clinic, both male and female	Clinical trial data analysed to determine if patients spontaneously cleared gonococcal infection	20.5% of patients analysed spontaneously cleared gonococcal infection; clearance was associated with genital, pharyngeal and rectal sites of infection; spontaneous clearance was not associated with patient demographics including previous gonococcal infection

Sera from female laboratory workers who volunteered serum, and had no history of gonococcal infection had no detectable antibodies with bactericidal activity against gonococcal strains isolated from patients with pelvic inflammatory disease (PID). In the same study, only 4 out of 13 men with uncomplicated gonococcal urethritis had bactericidal antibodies to the infecting gonococcal strain in their pretreatment serum. Uncomplicated infection of male subjects was described as men who presented with a urethral discharge from which *N. gonorrhoeae* was isolated. Of 9 women found to have gonococcal infection during a routine pelvic examination and recalled for treatment within 33 days, one had bactericidal antibodies in pre-treatment sera to the infecting isolate. 4 out of the 5 of these women for whom the interval between isolation of the bacteria and treatment was more than 33 days had bactericidal antibodies suggesting a correlation between prolonged asymptomatic carriage of gonococcus and the development of bactericidal antibodies. Furthermore, in patients with PID, the severity of illness correlated with the development of bactericidal antibodies (80). Complications arise in comparing studies, which often study the immune response to gonococcus in different states of gonococcal infection in both male and female subjects, which could lead to different conclusions. The mechanisms of different outcomes of gonococcal infection, from asymptomatic infection to ascending infection of the upper reproductive tract in women, has been extensively investigated (89).

In a separate study (81), 12 out of 13 patients with gonococcal infection had serum antibodies to Por (protein I), a gonococcal outer membrane antigen, detectable by Western blotting. 9 out of the 13 patients had serum antibodies to LOS, and 8 out of 13 patients had serum antibodies to both Por and LOS. All patients regardless of disease status had antibodies against at least one gonococcal antigen tested. Therefore, it is important to note the variability in human antibody response to gonococcus in terms of breadth of response to different antigens (80, 81). Functional antibody response is often variable and difficult to measure, since non-immune sera contain endogenous antibodies which can have a high background level of activity against gonococcus (90). The development of blocking antibodies which inhibit the function of protective antibodies in immune sera further complicates the picture (43). Correlations between serum bactericidal antibody levels and severe disease or length of infection (80) need to be confirmed in a larger study. A key question is the reason for lack of protective immunity following infection. This could either be an inherent failure to develop protective immunity or the result of lack of breadth of protection against other gonococcal isolates due to their high antigenic variation. As discussed, animal studies appear to show that *N. gonorrhoeae* inhibits the generation of adaptive immunity (72–74). However, it is not known if this work translates to humans, where variation of infecting strains complicates the situation.

**Figure 3:**
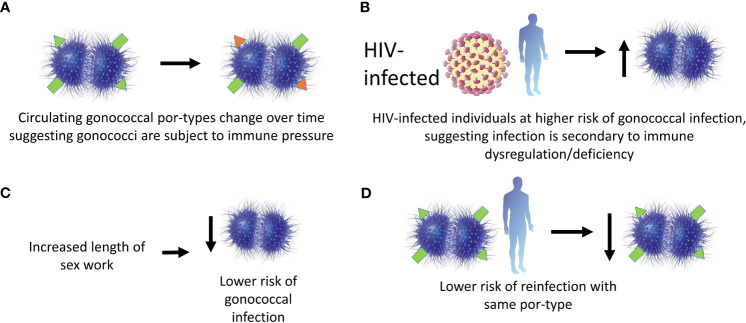
Evidence for protective immunity following gonococcal infection in a cohort of female commercial sex workers in Kenya. A cohort of 227 female sex workers in Kenya were followed in a longitudinal study over 16 months. Protective immunity in the cohort was said to be observed due to the findings supporting 4 hypotheses. These were: **(A)** the por-types of the infecting gonococci changed with time suggesting selective immune pressure; **(B)** HIV positive subjects were more likely to have gonococcal infections suggesting infection was associated with immune defects; **(C)** the likelihood of gonococcal infection decreased with the length of sex work suggesting a building-up of immunity with each exposure; and **(D)** subjects were unlikely to be re-infected with the same por-type suggesting at least por-type immunity is observed.

Recurrence of gonococcal salpingitis (inflammation of the fallopian tubes) is less likely with strains of the same Por (protein I) type, suggesting that Por-specific protective immunity can be induced, at least in the context of more disseminated infection (82). Por-type immunity was subsequently demonstrated in a larger study of 227 female sex workers in Nairobi, Kenya over 16 months, based on the testing of four hypotheses: 1. the prevalence of por-types making up the gonococcal population changed during the course of the study, which could have been driven by the development of protective immunity, 2. HIV-positive subjects (who were presumed to be immunodeficient) experienced more frequent gonococcal infections and were more at risk of reinfection, 3. the duration of prostitution correlated negatively with the frequency of infection (suggesting acquisition of immunity over time) and 4. for the majority of the serovars tested there was a reduced risk of reinfection with the same serovar (83). Therefore, there is epidemiological evidence for immunity to homologous gonococcal infection. A summary of the findings from this study is shown in **Figure 3**. Any immunity is apparently incomplete, since protection is observed only in the context of severe disease (82) or is Por-type specific (83). Further work is needed to establish the mechanistic basis for this incomplete protection.

Gonococcal antibody levels, both serum and mucosal, from male and female patients with gonococcal infection attending an STI clinic in Birmingham, Alabama, to formaldehyde-fixed gonococcal bacteria were shown to be only slightly higher than in uninfected individuals (84). However, if formaldehyde affects key gonococcal epitopes then this could underestimate the antibody response. In these patients, previous history of gonococcal infection did not affect levels of specific antibodies suggesting that immunological memory was not induced. Since these were uncomplicated gonococcal infections, it is possible that the lack of antibody response was due to the absence of inductive tissues (organized lymphoid tissue) at the site of relatively superficial infection. In female patients attending an STI clinic in Birmingham, Alabama, aged between 17 and 57, cytokine levels in genital secretions were not higher in individuals who had gonococcal infection compared with those that did not, while the level of serum IL-6 was elevated in patients with gonorrhea (85). Levels of IL-10 and TGF-β were also not higher in those with gonococcal infection, suggesting that a suppressive cytokine response may not be responsible for a lack of detectable pro-inflammatory cytokine response. This is further evidence for a relatively mild immune response to uncomplicated genital gonococcal infection in humans.

Therefore, for uncomplicated genital infection, the immune response is relatively weak (84, 85), which may explain why reinfection occurs frequently. As previously explained, it is possible to correlate immune response to more severe disease (82), suggesting that lack of protection resulting from uncomplicated infection could in part be due to a lack of immune induction. Moreover, there is evidence that protective immunity could be Por-type-specific (83) which would suggest a large role for antigenic variation as a reason for lack of protective immunity. The reality is possibly a combination of the two, since incomplete protective immunity has been demonstrated (83). Additionally, active immune evasion resulting from induction of anti-inflammatory cytokines by *N. gonorrhoeae* has been shown in the mouse model (72–74), but not yet in human studies. There is scope for more in-depth studies of cytokine levels in human patients, to expand the range of cytokines previously studied, in order to understand if, and to what extent, an immunosuppressive environment is induced.

In a study from 2011, the serum concentrations of IL-17A, IL-23 and IFN-γ were analyzed in 27 patients (consisting of 16 men and 1 woman, recruited from Rome and Turin, Italy, with median age of 37.5 years) with gonorrhea and compared to 17 healthy controls (86). The concentrations of all three cytokines were significantly higher in gonorrhea patients compared to controls. Furthermore, there was an inverse correlation between IFN-γ and IL-17A, suggesting an antagonistic relationship between Th1 and Th17 responses. Another study from 2015 examined women with STIs from Cape Town and Durban, South Africa, and found that those who had gonorrhea had higher levels of IL-17 at the genital site than controls who had no STIs, although the association between gonococcal infection and increased levels of IL-17 was not significant after accounting for co-infection with other STIs detected included *Chlamydia trachomatis*, *Trichomonas vaginalis*, HSV-2, bacterial vaginosis and candida infection (87). The cellular source of IL-17 during human gonococcal infection is still unknown.

A recent report studied 405 gonorrhea patients enrolled in a clinical trial to test the efficacy of gentamicin as a treatment for gonorrhea in the UK and included both women and men, heterosexuals and men-who-have-sex-with-men. The results demonstrated that spontaneous clearance of *N. gonorrhoeae* occurred in 20.5% of patients within a median of 10 days (88). Ethical concerns prevent these kinds of studies from being conducted for a prolonged time period, since treatment must commence following diagnosis. However, clearance was observed at different anatomical sites (genital, pharyngeal and rectal), and was not associated with the specific demographics of participants (88). This implies that in some cases an immune response is generated that clears infection. There was no association with previous history of gonococcal infection, so it is unlikely that clearance in those specific patients was due to previously-acquired immunity. Those who spontaneously cleared were less likely to have had dysuria, which may suggest a lower bacterial load during the infection. Further work is needed to understand the specific immune mechanisms behind spontaneous clearance.

### 4.2 Controlled human infection models

Controlled human infection models (CHIMs) involve the inoculation of gonococcus into the urethra of male volunteers (91). This allows for the characterization of infection dynamics, immune response and pathogenesis within the natural host. However, there are limitations. Only studies with male volunteers are permitted since the risk of serious disease from local spread of infection is much lower than in women. Furthermore, ethically the infection must be treated within a limited period of time or when clinical symptoms appear, restricting study length to around five days. Therefore, studies on the pathogenesis and immune response in more severe disease or chronic infection are not possible in the human challenge model.

Despite the limitations, CHIM studies have furthered understanding of the pathogenesis of *N. gonorrhoeae*. For example, antigenic variation of the pilus and phase variation of Opa have both been demonstrated during infection (92, 93). LOS phase variation also occurs (94) and sialylation of LOS decreases infectivity in volunteers (95). A mutant strain of gonococcus lacking *lptA*, which, when functional, adds phosphoethanolamine onto lipid A of LOS, showed reduced survival compared to the wild-type strain in a competition challenge experiment (96). Since *N. gonorrhoeae* is a human restricted pathogen, some facets of the ability of the bacteria to grow *in vivo* can only be studied in man. For example, a human challenge model was used to demonstrate that *N. gonorrhoeae* does not require the expression of IgA1 protease to infect male humans since the course of infection with a gonococcal strain deficient for this protease was indistinguishable from that of the wild-type strain (97).

CHIM studies have been used to assess the immune response to gonococcal infection. In an early study from 1969, 10 male volunteers recruited from Atlanta, Georgia, were infected with gonococcal strain F62 and the antibody response measured (98). 9 out of the 10 volunteers showed a 4-fold increase in IgG titer to unspecified heat-labile formalin-fixed gonococcal antigens. 7 of those 9 with an increase in IgG reached peak titers within 10 days of infection. There was a 4-fold increase in IgM and IgA to the same antigens in only 4 and 3 patients respectively. The number of subjects, was small, and the inoculum used [a full 2 mm bacteriological loop (99)] was higher than is likely during natural transmission, highlighting the general limitations of human challenge models.

Cytokine responses were measured in the urine of volunteers exposed to experimental infection with *N. gonorrhoeae* (100). 17 volunteers were infected and treated with antibiotics at the onset of symptoms or 7 days after challenge. 10 subjects went on to show symptoms, while 7 remained asymptomatic. IL-8 was present in the urine of all symptomatic subjects, and rapidly declined upon treatment. Similarly, IL-6 levels were elevated in all symptomatic volunteers, declined on treatment, and correlated with the number of viable gonococci detected and number of leukocytes recovered from the urine. An increase was also observed in IL-8 and IL-6 levels in 4 of the 7 volunteers who were asymptomatic, but these levels rapidly returned to baseline. All symptomatic subjects had elevated levels of TNF-α and IL-1β in the urine, while IL-1α and GM-CSF were consistently undetectable (100). The number of subjects with a 4-fold elevation in IL-8, IL-6, TNF-α or IL-1β in plasma compared with pre-challenge was more variable. Levels were already high pre-challenge. The majority of symptomatic subjects had elevated levels of IL-8 in plasma post-challenge, but only around half had elevated levels of TNF-α or IL-1β, and only 3 subjects had increased IL-6 levels. PBMC mRNA levels from the same study indicate that expression of TNF-α, IL-6 and IL-8 is not significantly altered during experimental infection, although the data were quite limited. The rapid increase of IL-8, IL-6 and TNF-α levels measured in the urine suggests that they are produced at the mucosal site, while a relative delay in IL-1β production suggests that this cytokine is derived from the infiltrating leukocytes. A more detailed study of the origins of these cytokines during human infection is needed as well as a study of a wider range of cytokines.

In a reinfection study in the US in 2001, 14 volunteers out of 15 were initially successfully infected with *N. gonorrhoeae* strain MS11mkC at a dose of 10^4^ CFU. Two weeks after treatment for the infection, the volunteers were re-challenged with a lower dose of 2.3 x 10^3^ CFU of the same strain. 6 of the 14 previously successfully infected volunteers were successfully reinfected and showed symptoms, as did 5 of 10 of a control naïve group (101). Therefore, no protection to reinfection was detected. A limitation with the CHIM studies to date is that only low numbers of volunteers have been included in each study. Therefore, these studies do not have sufficient power to detect low levels of protection. Only 2 of the 10 volunteers who were able to complete 7 days of infection without early treatment became re-infected at phase 2, suggesting a correlation between protection and length of infection, although there was not enough power in the study to observe a significant difference. However, induction of serum antibodies targeting LOS was associated with resistance to reinfection. It is still unclear how serum antibodies protect against *N. gonorrhoeae*. Protection could occur through translocation to the mucosa, or serum antibodies could be a surrogate marker for mucosal antibody responses. It may be that protection is cumulative with each gonococcal infection, boosting immunity with each encounter.

## 5 Prospects for a protective vaccine

There is no licensed vaccine for gonorrhea, and all vaccine candidates to date tested in efficacy studies in humans have failed (25, 102, 103). Field trials with a lysed whole cell vaccine candidate showed no efficacy compared to a placebo group (102). Similarly, volunteers vaccinated with purified gonococcal pilus were not protected against infection compared to a placebo arm (25).

A retrospective cohort study of individuals who had received an OMV vaccine against *N. meningitidis* serogroup B (MeNZB) in New Zealand found vaccine effectiveness of 31% (95% CI 21-39) in preventing a diagnosis of gonorrhea (10). Furthermore, MeNZB vaccination significantly protected against hospitalization from gonorrhea (104). Similarly, the incidence of gonorrhea in Cuba declined at the end of the 1990s following the introduction of an OMV vaccine against *N. meningitidis* (105). At the same time incidences of other STIs such as genital warts and syphilis were unaffected suggesting this was a specific effect on gonorrhea. Cases of gonorrhea also decreased in Quebec, Canada following mass vaccination with the 4CMenB, another licensed vaccine for *N. meningitidis* group B, which contains MeNZB OMVs as one of its components (12). A further matched cohort study in Southern California found that gonorrhea rates were 46% lower among recipients of 4CMenB than recipients of MenACWY (a polysaccharide conjugate vaccine against *N. meningitidis* serotypes A, C, W and Y, which does not contains OMVs) (106). 4CMenB vaccination has also been associated with a lower risk of gonorrhea in a case-control study of men-who-have-sex-with-men living with HIV (107). Even a relatively low efficacy vaccine against gonococcus could none-the-less have a significant impact on the population disease prevalence (108–111) and the above findings have brought renewed hope to developing a bespoke vaccine against gonococcus.

While the mechanism of cross-protection of 4CMenB against gonococcus is not clear, sera from individuals vaccinated with 4CMenB were shown to react with gonococcal proteins in ELISA and Western blots (112). Immunization with 4CMenB in the female mouse model of gonococcal infection has shown that vaccination significantly accelerated clearance and reduced bacterial burden (113). Serum IgG and vaginal IgA from the mice cross-reacting with gonococcal OMVs recognized several gonococcal proteins and had serum bactericidal activity against both the challenge strain F62 and strain FA1090. However, no correlates of protection have yet been identified. There was no correlation between bactericidal activity of mouse serum and clearance, and sera from humans immunized with 4CMenB did not show increased bactericidal activity compared with unvaccinated controls. At least one randomized controlled clinical trial is due to investigate the efficacy of 4CMenB against *N. gonorrhoeae* (114). The primary endpoint will be the number of gonococcal infections, while secondary endpoints include vaccine-induced immune responses. More work is needed to uncover the mechanisms of protection of 4CMenB against *N. gonorrhoeae* in humans, which could help understand correlates of protection and inform the development of gonococcal-based vaccines for *N. gonorrhoeae* infection. A summary of current knowledge about mechanisms of protection to gonococcal infection is presented in **Table 2**.

**Table 2:** Protective and non-protective immune mechanisms against gonococcal infection.

	Immune parameter	Mouse/human study	Evidence	Effect of parameter	Ref
Cellular immunity	Th17 response	Mouse model of infection	Blocking IL-17 leads to prolonged infection	Protective	(71)
	Th17 response	Mouse model of infection	IL-17 supresses protective Th1/Th2 responses	Inhibits protection	(72)
	TGF-β	Mouse model of infection	Blocking TGF-β led to accelerated clearance of gonococcus	Inhibits protection	(72)
	Th1/Th2 response	Mouse model of infection	Mice with gene deletions in IL-12 or IL-4 were impaired for clearing gonococcus compared to wild-type when TGF-β was blocked	Protective	(72)
	Th1 response	Mouse model of infection	Administration of encapsulated IL-12 leads to accelerated clearance of gonococcus	Protective	(75)
	IL-10	Mouse model of infection	Gonococcal infection stimulates IL-10 production, stimulating type 1 regulatory T cells. Blocking IL-10 leads to accelerated clearance of gonococcus	Inhibits protection	(74)
Antibody response	Serum bactericidal activity	Mouse model of infection	Protective effects of antibody mAb2C7 (anti-LOS) are lost in C1q and C9 null mice or when C5 function is lost	Protective	(79)
		Mouse model of infection	No correlation between bactericidal activity of mouse serum and clearance in mice vaccinated with 4CMenB	Non-protective	(113)
		Human retrospective cohort study	Serum from subjects immunised with partially protective 4CMenB did not show increased bactericidal activity over unvaccinated controls	Non-protective	(113)
	Anti-LOS antibodies	Controlled human infection model	In a model of reinfection, induction of serum anti-LOS antibodies was associated with resistance to reinfection	Protective	(101)
	Blocking antibodies	*In vitro*	Purified blocking IgG (from serum from a subject with disseminated gonococcal infection) blocked serum bactericidal activity of convalescent serum to gonococcus	Inhibits protection	(42)
		*In vitro*	Purified anti-Rmp antibodies blocked bactericidal activity. Serum depleted of anti-Rmp antibodies increased killing of gonococcus by immune serum	Inhibits protection	(43)
		Human retrospective cohort study	Subjects with anti-Rmp antibodies were at increased risk of infection	Inhibits protection	(44)
		Mouse model	Serum from mice immunised with Δ*rmp* gonococcus was more bactericidal than serum from mice immunised with wild-type gonococcus	Inhibits protection	(45)
		Mouse model	Passive transfer of anti-Rmp antibodies or immunisation with recombinant Rmp delayed clearance of gonococcus	Inhibits protection	(46)

Meningococcal OMVs accelerate clearance in the female mouse model of gonococcal infection, with OMVs made from a strain lacking PorA, PorB and RmpM stimulating the highest clearance rate (115). Evidence from this study suggests antibodies produced by these OMVs bind a wider range of targets than OMVs produced by wild-type strains. This is possibly because PorB is immunodominant but highly variable in gonococcus and so antibodies to PorB are likely not protective. Immune sera from mice immunized with these OMVs were used to identify target gonococcal antigens including PilQ, MtrE, NlpD and GuaB. Further work is required to discover the target antigens which lead to protective immunity to gonococcus. Proteomic methods have led to the discovery of a number of potential gonococcal antigens, with analyses mostly focusing on cell envelope proteins. One study identified five novel candidate antigens; BamA, LptD, TamA and the as yet uncharacterized NGO2054 and NGO2139 (116). These were ubiquitously expressed in different growth conditions, surface exposed and shown to elicit bactericidal antibodies that cross-reacted with a number of different gonococcal isolates. A further proteomic analysis of fifteen gonococcal strains reported nine novel vaccine candidates, based on conservation across most strains tested and localized to the cell envelope or outer membrane (117). While new vaccine candidates are being discovered, attention must be given to the heterogeneity of *N. gonorrhoeae*, and potential variation of antigens. A recent study compared the sequence variation of 34 gonorrhea antigens across more than 5000 clinical isolates. A single allele for eight antigens was represented in more than 80% of the isolates, while PorB and TbpB were highly variable. Thirteen candidate antigens were identified, based on high frequency distribution of a single allele, or low frequency sequence polymorphisms in surface loops (118). Potential vaccine antigens have also been discovered from *in vivo* studies. 36 targets were identified as expressed during human mucosal infection and predicted to be immunogenic, membrane-associated and conserved (119). Further studies will need to show that these proteins are immunogenic and drivers of functional immunity in either an animal model or in humans. A summary of gonococcal candidate antigens is shown in **Table 3**.

**Table 3:** Candidate gonococcal vaccine antigens.

Antigen	Description of Study	Reference
Pilus	A randomised, placebo-controlled, double-blind efficacy trial of a purified pilus vaccine was tested in men and women. Vaccinated volunteers developed an antibody response to pili from the vaccine strain and heterologous strains to a lesser extent. The vaccine failed to protect against gonococcal urethritis.	(25)
LOS 2C7 epitope	A peptide mimic of the 2C7 LOS epitope (MAP1) induced a Th1 response, bactericidal anti-LOS antibodies and accelerated clearance of gonococcus in a mouse model.	(78)
MetQ	Recombinant MetQ formulated with CpG induced antigen-specific serum and vaginal antibodies and accelerated clearance of gonococcus in a mouse model	(120)
TbpA/B	A hybrid antigen system consisting of foreign TbpB and neisserial TbpA accelerated clearance of gonococcus in a mouse model	(121)
	Investigation into the three dimensional structure of TbpA and effect of antibodies in blocking function of the protein.	(122)
PilQ, MtrE, NlpD, GuaB and others	Candidate antigens were identified using antisera from mice vaccinated with wild-type meningococcal OMVs and OMVs lacking PorA, PorB and RmpM	(115)
BamA, LptD, TamA, NGO2054, NGO2139	Candidate antigens were identified using proteomics. The candidates were ubiquitously expressed, surface exposed and elicited bactericidal antibodies that cross-reacted with a number of different gonococcal isolates	(116)
Candidate antigens identified from whole genome sequence analysis	Thirteen candidate antigens were identified by analysis of protein coding regions of whole-genome sequences of >5000 strains of *N. gonorrhoeae*. Candidates were highly conserved or had low frequency polymorphisms in exposed surface loops	(118)
Candidate antigens identified as expressed during human infection	Thirty six candidate antigens were identified as expressed during natural human mucosal infection, were predicted to be immunogenic, membrane-associated, and conserved	(119)

Knowledge of correlates of protection to gonococcal infection and disease is still lacking. This is largely due to the fact that natural infection doesn’t usually protect against reinfection. If this is due to a lack of inductive sites in the genital mucosa, to which gonococcal infection is often limited, it may be that a parenteral vaccine will induce protective immunity where natural infection cannot. Such a vaccine needs to improve on immunity stimulated by natural infection. The finding that *N. gonorrhoeae* induces the production of suppressive cytokines in mice (72–74), which can be overcome by vaginal administration of IL-12 (76) suggests that a vaccine should induce a Th1 polarized response in order to protect. However, it is not clear whether this holds true for a protective response in humans, although 4CMenB is formulated with alum as an adjuvant, which is Th2-promoting. Any vaccine candidate tested in mouse or human challenge studies should be used to understand better correlates of protection. It could be that repeat infections often occur because gonococcus is highly antigenically variable and so new infections could often occur with strains that are different from previously infecting strains. Another possibility is that any protective immunity is not long-lasting and wanes quickly. Clearly any successful vaccine would have to provide broad cross-protection against a number of distinct gonococcal strains, whilst having relatively long-lasting efficacy since boosters would not be desirable in LMICs, where the highest burden of gonococcal disease exists (2).

## 6 Summary

One of the main barriers to the development of a vaccine to *N. gonorrhoeae* is the lack of established correlates of protection. Reasons why previous vaccine efforts have failed are not well understood beyond the antigenic variation of the organism presenting an obstacle for broad protection. The establishment of a mouse model to investigate the immune response to gonococcus has provided information about the mechanisms by which *N. gonorrhoeae* evades immunity and prevents the generation of protective adaptive immunity. Studies in humans are critical due to the human-restricted nature of *N. gonorrhoeae* and have demonstrated the lack of protective immunity to natural infection with gonococcus, although reasons for this are not known to the same level of detail as in mice models.

Large cohort studies of naturally infected individuals have demonstrated varying degrees of protection gained from such infection. More work is needed to decipher the relevant functional antibody and T-cell responses to natural gonococcal infection in man. Human challenge studies come with drawbacks such as restriction to male volunteers and the short duration of study but have provided valuable information on the pathogenesis of the *N. gonorrhoeae*, as well as on the basic immune response to gonococcal infection. These models will be valuable going forward in providing a controlled environment for further investigations, such as whether protection can be achieved against a homologous second challenge, and to test responses to candidate vaccines. Such vaccines have to do better than nature in stimulating a protective response and have to provide protection against a broad-range of heterologous strains. Clinical trials investigating meningococcal serotype B vaccines for use against gonorrhea are beginning, summarised in **Table 4**. Nevertheless, a relatively low efficacy vaccine could have a significant impact on gonococcal infection and disease at a population level (108–111).

**Table 4:** Registered clinical studies assessing response to meningococcal serotype B vaccines against gonorrhea, found at ClinicalTrials.gov.

Title of Study	ClinicalTrials.gov ID	Sponsor	Location	Study Start	Study Completion	Description
Gonococcal vaccine study in key populations in Kenya (BexKPK)	NCT04297436	University of Oxford	Kenya	2021	2022	Assess humoral and T-cell responses elicited by 4CMenB against *N. gonorrhoeae*
Safety and efficacy study of meningococcal group B vaccine rMenB+OMV NZ (Bexsero) to prevent gonococcal infection	NCT04350138	National Institute of Allergy and Infectious Diseases	United States; Thailand	2020	2023	Phase 2 randomized, observer-blind, placebo-controlled, multi-site trial of 4CMenB
Efficacy study of 4CMenB (Bexsero) to prevent gonorrhoea (GoGoVax)	NCT04415424	Kirby Institute	Australia	2021	2025	Phase 3, double-blinded, randomized placebo-controlled, multi-centred trial evaluating the efficacy of 4CMenB in prevention of *N. gonorrhoeae* infection
Mucosal immunity against *Neisseria gonorrhoeae* after 4CMenB vaccination	NCT04722003	National Institute of Allergy and Infectious Diseases	United States	2021	2023	Phase 2 mechanistic clinical trial to assess the systemic and mucosal immunogenicity of 4CMenB against *N. gonorrhoeae*
Study to assess gonorrhea immune responses induced by a *N. meningitidis* vaccine	NCT04094883	University of North Carolina, Chapel Hil	United States	2019	2020	Test of whether 4CMenB induces immune responses to *N. gonorrhoeae*
A study to learn about how Trumenba vaccine shots work against gonorrhea infection in teenagers and young adults in the United States	NCT05873751	Pfizer	United States	2023	2023	Retrospective cohort study of subjects who have received the meningococcal serotype B vaccine Trumenba
Efficacy of immunizion with 4CMenB in preventing experimental urethral infection with *Neisseria gonorrhoeae*	NCT05294588	University of North Carolina, Chapel Hill	United States	2022	2028	Double-blind, randomized controlled trial to test the efficacy of 4CMenB in preventing *N. gonorrhoeae* infection in a controlled human challenge model
Immunization for adolescents against serious communicable diseases (B part of it NT)	NCT04398849	University of Adelaide	Australia	2021	2024	Observe effectiveness of 4CMenB against gonococcal carriage
Efficacy trial on meningococcal B vaccine for preventing gonorrhea infections	NCT05766904	Chinese University of Hong Kong	Hong Kong	2023	2025	Randomized double-blind placebo-controlled trial testing the efficacy of 4CMen B on incidence of gonorrhea in men-who-have-sex-with-men

## Author contributions

TB wrote the first draft of the manuscript. All authors contributed to the article and approved the submitted version.
